# Evidence of Pepsin-Related Ocular Surface Damage and Dry Eye (PROD Syndrome) in Patients with Laryngopharyngeal Reflux

**DOI:** 10.3390/life10090202

**Published:** 2020-09-15

**Authors:** Rocco Plateroti, Marta Sacchetti, Giuseppe Magliulo, Andrea Maria Plateroti, Annalisa Pace, Antonietta Moramarco, Alessandro Lambiase, Alice Bruscolini

**Affiliations:** 1 Department of Sense Organs, Sapienza University of Rome, 00161 Rome, Italy; rocco.plateroti@uniroma1.it (R.P.); marta.sacchetti@uniroma1.it (M.S.); giuseppe.magliulo@uniroma1.it (G.M.); annalisa.pace@uniroma1.it (A.P.); antonietta.moramarco@uniroma1.it (A.M.); alice.bruscolini@uniroma1.it (A.B.); 2 NESMOS Department, S. Andrea Hospital, Faculty of Medicine and Psychology, Sapienza University of Rome, 00189 Rome, Italy; andreamaria.plateroti@uniroma1.it

**Keywords:** laryngopharyngeal reflux, ocular surface, ocular discomfort symptoms, dry eye, lacrimal pepsin levels

## Abstract

Background: patients with laryngopharyngeal reflux (LPR) showed detectable levels of tear pepsin that explain the nasolacrimal obstruction. The purpose of this study was to determine whether patients with LPR show ocular surface changes and to investigate the relationship between lacrimal pepsin concentration and ocular alterations. Methods: Fifty patients with positive endoscopic signs for LPR and an equal or higher score of 13 and 7 for Reflux Symptom Index and Reflux Finding Score were enrolled. Twenty healthy patients with no reflux disease and dry eye were included as the control group. After evaluation of ocular discomfort symptoms, the tear break-up time test, corneal staining, and tear sampling were performed. Tear pepsin levels were measured using Pep-test^TM^ kit. Results: Patients with LPR showed ocular surface changes including epithelial damage (48%) and impairment of lacrimal function (72%). Tear pepsin levels were detectable in 32 out of 50 (64%) patients with LPR (mean ± SD: 55.4 ± 67.5 ng/mL) and in none of the control subjects. Most of the LPR patients complained of ocular discomfort symptoms, including itching (38%), redness (56%), or foreign body sensation (40%). Tear pepsin levels were significantly correlated with the severity of LPR disease and with ocular surface changes. Conclusions: A multidisciplinary approach, including ophthalmological evaluation, should be considered in order to improve the management of patients with LPR.

## 1. Introduction

Laryngopharyngeal reflux (LPR) is a disease characterized by retrograde reflux of gastric and/or duodenal contents through the upper esophageal sphincter, causing an inflammatory reaction of the larynx, oropharynx, and/or nasopharynx. [1] The incidence of LPR is estimated between 4% to 10% in the general population [2,3]. The most frequent symptoms of LPR are represented by dysphonia, chronic cough, throat inflammation, and pharyngeal globe [4,5,6]. Endoscopic evaluation in patients with LPR shows laryngeal edema and hyperemia associated with granulomatous and polypoid lesions [3]. These changes have been related to an increase of pepsin concentration in the upper respiratory tract structures in patients with LPR [7,8,9]. Pepsin is a proteolytic enzyme produced by the gastric mucosa during digestive activity [10]. In the physiological condition, pepsin is detectable only in the stomach, while in patients with LPR, the presence of pepsin was demonstrated also in the salivary fluid, and salivary pepsin dosage was proposed as a biomarker for LPR diagnosis [11,12]. In addition, it has been shown that pepsin can reach other nasopharyngeal-related structures during reflux episodes, such as the middle ear in patients with otitis media or nasal cavity of patients with chronic rhinosinusitis [13,14,15,16,17,18,19]. Pepsin has been detected also in the tear film of patients with gastroesophageal reflux (GER) and in children with LPR [20,21]. Recently, the presence of ocular discomfort symptoms has been associated with the suspect of LPR [22]. However, the development of ocular surface changes and the presence of pepsin in tears in adult patients with LPR have not yet been evaluated.

The aim of this study is to evaluate ocular surface alterations in adult patients with LPR and the relationship with tear pepsin levels.

## 2. Materials and Methods

### 2.1. Patients Recruitment

This study was performed at the Department of Sense Organs of Sapienza University of Rome and patients with signs and symptoms of LPR were enrolled consecutively between October 2017 and June 2018.

Patients with LPR have been enrolled by an otorhinolaryngologist with the following inclusion criteria: aged older than 18 years, no-smokers, with positive endoscopic signs for LPR, and an equal to or higher score of 13 and 7 for the Reflux Symptom Index (RSI) and Reflux Finding Score (RFS), respectively. We excluded patients affected by autoimmune or infectious diseases and in treatment with pump inhibitors or other drugs used for the LPR treatment at the time of the study. Twenty healthy subject, sex- and age-matched, were recruited as a control group by an ophthalmologist and were referred to otorhinolaryngologist for LPR evaluation. All smokers with reflux disease and/or ocular surface disease including dry eye were excluded from the control group.

### 2.2. Ethics Statements

The research was conducted in accordance with the ethical principles of the Declaration of Helsinki and it was approved by the Institutional Review Board of Sapienza University, Rome, Italy, (identification code is 4841 prot n.14/18). Written informed consent, including approval for the use of information collected during the study, was obtained from the participants.

### 2.3. Otorhinolaryngology Evaluation

Patients were evaluated for the presence of LPR by complete clinical history collection, including evaluation of RSI and RFS.

RSI is a self-conducted questionnaire based on nine items, evaluating the presence of reflux symptoms. The test was considered positive when the score was ≥13 [23]. A validated Italian version of the RSI questionnaire, as described by Schindler, was used in the study [24].

All patients were evaluated by endoscopy by the same physician using a flexible endoscope connected to a camera and a high-definition monitor (Full HD) and RFS was assessed. Specifically, RFS evaluates the presence of 8 laryngoscopy findings with a scale between 0 (normal) to 26 (strongly pathological). RFS higher than 7 was considered pathological and indicative of LPR [25]. Patients with both RSI and RFS positivity were classified as clinically positive for LPR.

### 2.4. Ophthalmological Evaluation

All patients and healthy subjects were evaluated by an ophthalmologist for the presence of ocular discomfort symptoms, including redness, foreign body sensation and itching. Slit lamp examination was performed by the same physician in order to evaluate signs of ocular surface alterations, including conjunctival hyperemia and superficial punctate keratitis (SPK). Specifically, conjunctival hyperemia was scored from 0 (absent) to 2 (severe hyperemia) and SPK was evaluated by fluorescein vital staining and scored by Oxford grading Scale [26]. The tear film stability was assessed using the Break Up Time test (BUT). The ophthalmologist was blinded to the results of the otorhinolaryngology tests and to the questionnaires’ scores.

### 2.5. Tear Collection and Pepsin Evaluation

A collection of 100 microliters of tear samples were performed in the early hours of the morning in both eyes from all patients with a micropipette, a silicone tube siliconized to a small tank provided with a suction tube. The micropipette works by suctioning the tear fluid from the lacrimal lake, at the level of the inner canthus of the eyelid. The tears of both eyes were harvested and carried in a single test tube.

The collected tears were analyzed by Pep-test^TM^ kit (BIOHIT HealthCare, Milan, Italy), that is a qualitative and quantitative test to dose the pepsin concentration in body fluids. The test required 100 µL of tears with the addition of 100 µL of 0.01 M citric acid. Each sample was centrifuged at 400 rpm for 5 min. Subsequently, 80 µL of supernatant was collected and was added to 240 µL of migration buffer, and the mixture was vortexed for 10 s: 80 µL of this mixture was pipetted into the well of the Pep-test^TM^ Lateral Flow Device (LFD) and the results were ready after 15 min.

The test is based on a chemical reaction antigen-antibody utilizing a monoclonal anti-pepsin antibody (T band reveals the pepsin presence). In addition, the system involves an inner reaction control useful for estimating the system’s integrity (C band). The test is considered valid when both bands are obtained. The concentration of pepsin level was accurately measured by the Pep-test Cube that displays the result directly in ng/mL. The Pep-test is able to detect a minimum amount of pepsin equal to 16 ng/mL by a colorimetric test.

### 2.6. Statistical Analysis

Statistical analysis was performed using SPSS software version 22.0 (IBM, Armonk, NY, USA). Normal distribution of data was assessed by Shapiro-Wilk test and independent sample T-test was used to compare tear levels of pepsin and clinical and demographic variables between groups. The Fisher exact test was used to evaluate the association between the presence of symptoms and signs and tear pepsin. Correlation between RSI/RSF score, ocular surface changes, and pepsin concentration were performed using the Spearman rho test. A value of *p* < 0.05 was considered statistically significant.

## 3. Results

Fifty patients affected by LPR (21 male and 29 female; mean age: 41.2 ± 15 years) and 20 healthy subjects (11 male and 9 female; mean age: 41.8 ± 8.5 years) were enrolled in this study. Both groups were homogeneous for gender and age and their clinical and demographical characteristics are described in Table 1.

Ocular evaluation demonstrated that patients with LPR showed ocular surface modifications when compared with healthy subjects (Table 1). Specifically, most of patients with LPR complained with at least one ocular discomfort symptom, including itching (38%), redness (56%) or foreign body sensation (40%) and showed SPK (48%) and/or impairment of tear film stability (72%) (Table 1).

Thirty-two out of 50 (64%) of patients with LPR showed detectable pepsin values in tears (mean ± SD: 55.4 ± 67.5 ng/mL), whereas in the control group lacrimal pepsin was undetectable in all subjects (*p* < 0.001).

**Figure 1:**
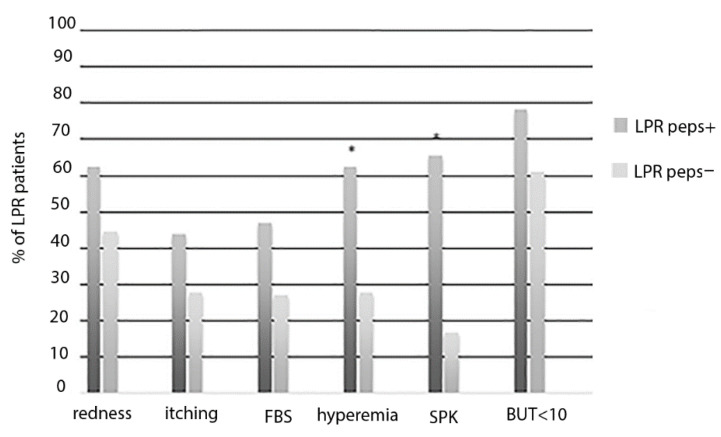
Percentage (%) of LPR patients with signs and symptoms of ocular surface impairment in the presence (LPR peps+) or absence (LPR peps−) of lacrimal pepsin concentration. FBS, foreign body sensation; SPK, superficial punctate keratitis; BUT, break-up time. * Represent statistical significance LPR peps+ versus LPR peps+.

The pepsin tear concentration was significantly correlated with LPR severity scores, namely, RSI (R = 0.370, *p* = 0.002) and RSF (R = 0.338, *p* = 0.004), and the severity of ocular surface changes in patients with LPR. Specifically, the higher pepsin levels in tears were significantly correlated with the higher severity of conjunctival hyperemia (R = 0.681, *p* < 0.001) and the Oxford Scale (R = 0.702, *p* < 0.001). The higher levels of lacrimal pepsin were also significantly correlated with lower tear film stability values assessed using the BUT test (R = −0.563, *p* < 0.001). Patients with LPR and detectable levels of lacrimal pepsin (LPR peps+) were compared with patients with LPR and absence of tear pepsin (LPR peps−). A higher number of patients with LPR peps+ showed the presence of hyperemia and SPK when compared with LPR peps− patients (Figure 1).

Specifically, LPR peps+ patients showed a significant increase of hyperemia score (0.7 ± 0.6 vs. 0.2 ± 0.4; *p* = 0.006) and Oxford score (0.7 ± 0.6 vs. 0.4 ± 0.1; *p* = 0.003) and a significant decrease of BUT (LPR peps+: mean ± SD 7 ± 2.8 versus LPR peps−: 9.5 ± 2.3; *p* = 0.005) when compared with LPR peps− patients.

As showed in Table 2, levels of lacrimal pepsin were significantly higher in the presence of signs and symptoms of ocular surface disorders, including itching, foreign body sensation, conjunctival hyperemia, SPK and abnormal BUT.

## 4. Discussion

In this study, we demonstrated that more than half of patients with LPR complain of ocular discomfort symptoms associated with a reduction of tear film stability and epithelial damage. These findings are in line with the previous evidence of an extra-esophageal involvement in patients with LPR including the mouth and ear [22,27,28,29,30,31]. Similarly to other mucosa lesions reported in LPR patients, ocular surface changes may be related to inflammatory and/or irritating factors such as pepsin [32].

In line with this hypothesis, pepsin was present in the tears of 64% of patients with LPR, while lacrimal pepsin was undetectable in all the healthy subjects. In addition, the higher pepsin tear levels were significantly correlated with the higher severity scores of LPR, confirming the role of pepsin as a biomarker of this condition [21]. The presence of pepsin in tears of LPR patients has not yet been explained. Magliulo et al. hypothesized a mechanical mechanism in which pepsin crosses the nasopharynx during the reflux attack, and reaches the tear film through the nasolacrimal duct [20]. However, an active secretion of pepsin in tears may also be hypothesized as a response to the acid-induced stimulation of tear parasympatic reflex. Alternatively, pepsin may be released during ocular surface inflammatory reaction [33].

Accordingly, higher levels of lacrimal pepsin levels were associated with higher severity of ocular symptoms, increased conjunctival hyperemia, higher Oxford score, and reduced tear film stability. This evidence suggests that the local increase of pepsin concentration could affect ocular surface structures and functionality though its proteolytic action and/or inflammatory stimulation. It has been clearly demonstrated that pepsin is active at an acidic pH which can be found at the ocular surface during inflammation; however, in the larynx, pepsin can also be endocytosed and activated in the lysozymes [34]. How this enzyme could damage ocular surface epithelia may only be hypothesized based on the evidence described in other mucosal lesions [35,36]. Specifically, the proteolytic activity of pepsin could directly affect the ocular surface, causing epithelial damage associated with hyperemia and irritative symptoms, such as foreign body sensation. Otherwise, a previous study showed increased levels of inflammatory cytokines in pepsin treated human hypopharyngeal epithelial cell in vitro [37]. Based on these findings, the presence of pepsin on the ocular surface may induce the expression of several proinflammatory cytokines leading to symptoms and signs of keratoconjunctivitis. Moreover, it has been demonstrated that pepsin acts as mucolytic agent and mucin gene regulator in digestive mucosa [38,39]. Similarly, it is possible that lacrimal pepsin modifies ocular surface mucin layer, inducing changes of the tear film stability and reducing ocular surface protection with consequent epithelial damage and inflammation.

These results suggest a pathogenetic role of lacrimal pepsin in the development of ocular surface alteration. However, ocular environmental alteration, such as tear instability, changes of pH, and inflammatory reaction, may also play a role in this condition. A novel ocular clinical entity, characterized by pepsin related ocular surface damage and dry eye, named PROD syndrome, may be proposed to describe ocular surface changes in LPR patients.

Several studies suggest that pepsin can be used as a reliable marker for the diagnosis of LPR; however, the accuracy, timing, and threshold values of pepsin test remain open questions [40,41]. Recently, a prospective study proposed a salivary pepsin cut-off value as an alternative tool for diagnosis of LPR [42]. The role of lacrimal pepsin test should be further investigated in a prospective study with a larger population of LPR patients included with the gold standard tests (pH throughout 24 h monitoring), in order to verify the role of pepsin as an additional tool for LPR diagnosis and its role in PROD syndrome.

In conclusion, a multidisciplinary approach of LPR disease, including ophthalmological evaluation, should be performed in LPR patients in order to identify the presence of ocular surface changes and to improve the management of these patients.

## Figures and Tables

**Table 1:** Clinical and demographical changes of the study population.

Variable	LPR+ (*n* = 50)	Control Group (*n* = 20)	*p* Value
Age, y			
Mean (SD)	41.2 (15)	41.8 (8.5)	0.835
Sex Number			
M	21	11	
F	29	9	0.235
Otorhinolaryngology evaluation			
RSI score			
Range	13–33	3–12	
Mean (SD)	21.4 (6.3)	7.4 (2.7)	<0.001 *
RSF			
Range	8–17	2–6	
Mean (SD)	11.2 (2.6)	3.9 (1.3)	<0.001 *
Ophthalmological evaluation			
Redness Number (%)	28 (56)	2 (1)	0.001 *
Itching Number (%)	19 (38)	1 (0.5)	0.004 *
FBS Number (%)	20 (40)	2 (1)	0.019 *
SPK Number (%)	24 (48)	0	<0.001 *
Conjunctival hyperemia			
Number (%)	25 (50)		
Score, Mean (SD)	0.5 (0.6)	0 (0)	<0.001 *
Oxford			<0.001 *
Score, Mean (SD)	0.50 (0.6)	0 (0)	
BUT			
Mean (SD)	7.8 (2.9)	13.2 (1.7)	
BUT < 10 s Number (%)	36 (72)	0	0.005 *

RSI: Reflux Symptom Index; RFS: Reflux Finding Score; FBS: foreign body sensation; SPK: superficial punctate keratitis; BUT: break-up time. * statistically significant *p* < 0.05

**Table 2:** Pepsin concentration in patients with LPR (*n* = 50) in the presence (YES) and absence (NO) of signs and symptoms of ocular surface disorders.

Variable	Tear Pepsin Concentration Mean ± SD (ng/mL)	*p* Value
Redness		
YES	70.1 ± 76.3	
NO	40.4 ± 50.9	0.113
Itching		
YES	98.3 ± 79.9	
NO	28.9 ± 39.7	
FBS		
YES	98.8 ± 76.2	
NO	39.3 ± 52.7	0.01 *
Conjunctival hyperemia		
YES	112.2 ± 67	
NO	19.2 ± 26.1	<0.001 *
SPK		
YES	106.1 ± 65.9	
NO	19.2 ± 29.3	<0.001 *
BUT < 10 s		
YES	70.2 ± 73.9	
NO	29.8 ± 43.9	0.042 *

FBS: foreign body sensation; SPK: superficial punctate keratitis; BUT: break-up time; * statistically significant *p* < 0.05.
